# Novel cationic peptide TP359 down-regulates the expression of outer membrane biogenesis genes in *Pseudomonas aeruginosa:* a potential TP359 anti-microbial mechanism

**DOI:** 10.1186/s12866-016-0808-2

**Published:** 2016-08-22

**Authors:** Ejovwoke F. Dosunmu, Atul A. Chaudhari, Swapnil Bawage, Mona K. Bakeer, Donald R. Owen, Shree R. Singh, Vida A. Dennis, Shreekumar R. Pillai

**Affiliations:** 1 Center for NanoBiotechnology Research, Alabama State University, Montgomery, AL USA; 2 LSU Health Sciences Center, School of Allied Health Professions, New Orleans, LA USA; 3 Therapeutic Peptides Inc., Baton Rouge, LA USA

**Keywords:** *Pseudomonas aeruginosa*, Antimicrobial peptides, Antimicrobial effect

## Abstract

**Background:**

Antimicrobial peptides (AMPs) are a class of antimicrobial agents with broad-spectrum activities. Several reports indicate that cationic AMPs bind to the negatively charged bacterial membrane causing membrane depolarization and damage. However, membrane depolarization and damage may be insufficient to elicit cell death, thereby suggesting that other mechanism(s) of action could be involved in this phenomenon. In this study, we investigated the antimicrobial activity of a novel antimicrobial peptide, TP359, against two strains of *Pseudomonas aeruginosa*, as well as its possible mechanisms of action.

**Results:**

TP359 proved to be bactericidal against *P. aeruginosa* as confirmed by the reduced bacteria counts, membrane damage and cytoplasmic membrane depolarization. In addition, it was non-toxic to mouse J774 macrophages and human lung A549 epithelial cells. Electron microscopy analysis showed TP359 bactericidal effects by structural changes of the bacteria from viable rod-shaped cells to those with cell membrane damages, proceeding into the efflux of cytoplasmic contents and emergence of ghost cells. Gene expression analysis on the effects of TP359 on outer membrane biogenesis genes underscored marked down-regulation, particularly of *oprF*, which encodes a major structural and outer membrane porin (OprF) in both strains studied, indicating that the peptide may cause deregulation of outer membrane genes and reduced structural stability which could lead to cell death.

**Conclusion:**

Our data shows that TP359 has potent antimicrobial activity against *P aeruginosa*. The correlation between membrane damage, depolarization and reduced expression of outer membrane biogenesis genes, particularly *oprF* may suggest the bactericidal mechanism of action of the TP359 peptide.

**Electronic supplementary material:**

The online version of this article (doi:10.1186/s12866-016-0808-2) contains supplementary material, which is available to authorized users.

## Background

With continuous usage of antibiotics overtime, pathogenic bacteria buildup resistance, and this is a major drawback for the treatment of a plethora of bacterial infections. Hence, there is an increasing need for antimicrobial agents with novel mechanisms of action, which target essential cellular metabolic processes and pathways. In order to combat recalcitrant pathogens, essential targets for viability, growth and pathogenicity must be identified [1].

Antimicrobial peptides (AMPs) are a class of antimicrobial agents that have been of interest, and they are considered to be a potential substitute for conventional antibiotics [2]. AMPs are a group of bactericidal molecules of the innate immune system in multicellular organisms and in unicellular organisms, represses competing species [3]. Cationic AMPs are small positively charged amphipathic peptides; having both hydrophilic and hydrophobic ends, which enable the molecules to be soluble in aqueous medium and to penetrate lipid-rich membranes of bacterial cells. It is well established that electrostatic attraction between the positively-charged antimicrobial peptides (+2 to +9) [4] and the highly negative-charged bacterial membrane enables the first step of attachment of the peptide to the bacteria [5]. The negative charge is due to composition of the membranes of bacteria, which is different from eukaryotic cells. This difference partially explains the sensitivity of bacterial cells to AMPs and the lack of toxicity to eukaryotic cells [6].

Although the exact mechanisms of action of AMPs are not fully understood, several have been proposed, such as the interaction of AMPs with membranes in the carpet, toroid and barrel-stave models [7] causing membrane damage. Additionally, other mechanisms of action have been attributed to the integration of AMPs into the cytoplasmic membrane. However, membrane perturbation/depolarization alone may not be sufficient to cause cell death by cationic AMPs, as reported for gramicidin S action against *P. aeruginosa* [8]. Furthermore, there is a gap in studies addressing the interaction of AMPs with essential genes involved in cellular metabolism and membrane biosynthesis genes.

*P. aeruginosa* is a ubiquitous Gram-negative bacterium and one of the most important clinical opportunistic pathogens. It is a persistent pathogen associated with nosocomial infections [9], wound infections [10, 11], urinary tract infections (UTIs) [12], otitis media, nasal infections [13] acute and chronic lung infections in artificially ventilated [14] and cystic fibrosis (CF) patients [15, 16]. *P. aeruginosa* infection is difficult to eradicate completely, especially in CF patients, in whom, infection results in decreased lung function and uncontrolled inflammation [17, 18]. It continues to pose a therapeutic problem due to drug resistance developing during therapy, caused by low permeability of its outer membrane, multi-drug efflux pumps and AmpC β-lactamases [19] resulting in high rates of morbidity and mortality. Additionally, during the initial colonization of *P. aeruginosa*, the non-mucoid phenotype is the dominant phenotype, however, as the infection progresses there is a switch to a more persistent mucoid phenotype [20]. This conversion results from the production of a large quantity of alginate [21], which is followed by the formation of a protected biofilm of micro-colonies [15, 22, 23], resulting in persistent infections [24], poor prognosis of infected patients and mortality and morbidity in CF patients. The mucoid state also enables *P. aeruginosa* to elude opsonization, phagocytosis, and digestion by host defense mechanisms [25].

In this study, the antimicrobial activity of a novel antimicrobial peptide TP359 was investigated against a mucoid and a non-mucoid strain of *P. aeruginosa.* We assessed its effect on membrane biogenesis, peptidoglycan-associated genes and on essential genes of *P. aeruginosa* in order to determine the possible mechanisms of action of AMPs.

## Methods

### Bacterial cultures and reagents

*P. aeruginosa* strains ATCC 39324 and ATCC 27318 used in this study were purchased from the American Type Culture Collection (Manassas, VA). Cation-adjusted Mueller Hinton broth (CA-MHB; Becton Dickson, Franklin Lakes, NJ) was used to grow the bacteria for determination of in-vitro antimicrobial activity and time-kill assays. Luria-Bertani medium (LB; Becton Dickson) was used for the membrane-depolarization assay and quantitative real time-polymerase chain reaction (qRT-PCR) analysis. Todd-Hewitt medium (Becton Dickson) supplemented with yeast extract (THY) was used to grow the bacteria for scanning electron microscopy (SEM) and transmission electron microscopy (TEM). Gentamicin sulphate and polymyxin B were purchased from Fisher Scientific (Pittsburgh, PA).

### Antimicrobial peptide TP359

The proprietary antimicrobial peptide (AMP) TP359 (MYR-KKALK-[K]D-amide, C_41_H_81_N_9_O_6_) was synthesized with > 95 % purity by Therapeutic Peptides Inc., (Baton Rouge, LA). The structure of TP359 was predicted de novo by converting the peptide sequence to its tertiary structure using PEPStr; a method for tertiary structure prediction of small bioactive peptides [26]. TP359 was one of several proprietary AMPs from Therapeutic peptides Inc. that was screened for antimicrobial activity against *P. aeruginosa* (data not shown). TP359 showed enhanced antimicrobial activity against *P. aeruginosa* and thus was selected for further studies.

### Bactericidal assay

We evaluated the bactericidal activity of TP359 against *P. aeruginosa* by first determining the minimum inhibitory concentration (MIC). TP359 was serially diluted 2-fold starting at 64 to 0 μg/mL and added into non-treated polystyrene 96-well plates, according to the Clinical and Laboratory Standards Institute (CLSI) M7-A7 method [27] as previously described [28]. Each well was next inoculated with 20 μL (1 × 10^6^ CFU/mL) of either strain of *P. aeruginosa* in CA-MHB to a final concentration of approximately 1 × 10^5^ CFU/mL. Bacteria alone served as positive control to verify bacteria growth, while negative control wells contained only media to ascertain culture sterility. To determine the minimum bactericidal concentration (MBCs) [29] we used TP359 at two or three concentrations higher than its MIC. The bacteria concentrations were then plated on Luria agar (LA) plates and viable CFU/mL counts were enumerated by plate count assay.

### Time-kill studies

The time-kill studies of TP359 against both strains of *P. aeruginosa* were performed as we recently reported [28], according to the M26-A guidelines of the CLSI [30]. Either *P. aeruginosa* strains (1 × 10^5^ CFU/mL) were inoculated into non-treated polystyrene 96-well plates containing TP359 concentrations correlating to their respective MICs (0.5×, 1×, 2×, 4× and 8× MIC). Viable cell counts were determined after 2, 4, 8 and 24 h of incubation at 37 °C by plate count assay.

### Toxicity of the peptide to eukaryotic cell lines

Cell lines used in this study were the human lung carcinoma epithelial A549 cell line (ATCC® CCL-185™) and the mouse J774 macrophage cell line (ATCC® TIB-67™). A549 was used because *P. aeruginosa* is known to infect the respiratory tract of cystic fibrosis (CF) patients, while macrophages are the first line of defense during infection. The A549 and J774 cell lines were cultured in F-12K Medium (Life Technologies, Grand Island, NY) and Dulbecco’s Modified Eagle Medium (DMEM, Life technologies), respectively supplemented with 10 % fetal bovine serum (FBS; Life technologies) and 1 % 100× antibiotic-antimycotic (Life technologies). The cytotoxic effect of TP359 on cell lines (A549 and J774) was determined using the Cell-Titer 96® Non-Radioactive Cell Proliferation assay kit (Promega G4000, Madison WI) as previously described [31].

### Membrane permeabilization assay

The effect of TP359 on outer membrane integrity of *P. aeruginosa* was analyzed using the LIVE/DEAD Baclight^TM^ viability kit L13152 (Molecular probes, Life Technologies) according to the manufacturer’s protocol. Overnight cultures of bacteria were inoculated into THY and grown at 37 °C to an optical density of 0.1 (1 × 10^9^ CFU/mL) before being exposed to 4× MIC for 4 h. Negative cultures included those without added peptide. Cultures were centrifuged at 2000 × g for 10 min, washed, further incubated in 1 mL of 0.85 % NaCl for 1 h and then finally re-suspended in 200 μL of 0.85 % NaCl. Bacteria cells were then incubated in the dark for 45 min in a 2X stock solution of the baclight staining reagent containing a final concentration of 6 μM SYTO 9 and 30 μM of propidium iodide. Images were captured using a Nikon Eclipse TE200 microscope (Nikon, Melville, NY, USA) using FITC-HYQ (Ex 450–500) and TRITC HYQ (Ex 530–550) filters.

### Membrane depolarization assay

Cytoplasmic membrane depolarization was determined using the fluorescent dye 3,3-dipropylthiacarbocyanine iodide [DiSC_3_(5); Fisher Scientific, Waltham MA] as described [32] with some modifications. Exponential-phase bacteria at approximately 1 × 10^9^ CFU/mL in buffer (5 mM HEPES, 5 mM glucose; pH 7.4) were incubated in 0.4 mM DiSC_3_ (5). After this, KCl (100 mM) was added to equilibrate the cytoplasmic and external K^+^ concentration followed by addition of bacterial suspension with either peptide, polymyxin B or gentamicin sulphate to optiplate 96-well white plates (Fisher Scientific) for concentrations ranging from 10 to 80 μM. The fluorescence intensity was measured by using the Hidex Plate Chameleon V (Lablogic Systems, Brandon FL) with an excitation wavelength of 622 nm and emission wavelength of 670 nm.

### Scanning and transmission electron microscopy analysis (SEM and TEM)

SEM and TEM analyses of untreated- and TP359-treated exponential-phase bacteria (1 × 10^9^ CFU/mL) were performed essentially as previously described [28]. Bacteria were exposed to approximately 4× MIC for 4 h at 37 °C prior to analyses.

### Peptide effect on essential genes of *P. aeruginosa*

To determine the effect of TP359 on *P. aeruginosa* genes essential for viability, cellular metabolism, pathogenicity, outer membrane biosynthesis and assembly (Table 1), were analyzed using qRT-PCR under bacteriostatic and bactericidal conditions. The bacteriostatic condition is indicated by bacterial recovery after 18 h of TP359 treatment, whereas at the bactericidal condition which causes cell death; samples were treated for a shorter time, to avoid complete cell death. *P. aeruginosa* at 1 × 10^5^ CFU/mL were exposed to 0.5× MIC of TP359 and grown for 18 h in a shaker incubator at 37 °C to evaluate the bacteriostatic effects of TP359. For the bactericidal condition, *P. aeruginosa* at 1 × 10^9^ CFU/mL were exposed to 4× MIC of TP359 and grown for 4 h, and subsequently for 10 and 30 mins to compare the effect at these time period to the bacteriostatic 4 h treatment condition. Also, *​P. aeruginosa* at 1 × 10^9^ CFU/mL were exposed to 4× MIC of gentamicin and grown for 4 h for control. Total RNA samples from both conditions were purified using an RNeasy Mini kit (Qiagen, Valencia, CA), and quantified using a spectrophotometer (Nanodrop 2000c, ThermoFisher Scientific, Waltham, MA). The cDNA synthesis was carried out using the Applied Biosystems High Capacity cDNA Reverse Transcriptase Kit (Life Technologies). One microgram of RNA was used to amplify selected key outer membrane biogenesis (*oprI*, *oprL*, *oprF*, *acpP*, *uppS* and *opr86*), cell division (*ftsA* and *ftsZ*) and virulence (*ampR* and *lasR*) genes of *P. aeruginosa* using the Applied Biosystems ViiA 7TM real time PCR system (Life Technologies). Primer pairs for each gene are shown in Table 2. The amplification conditions used were one cycle of initial denaturation at 95 °C for 2 min, followed by 40 cycles of 95 °C for 15 s, 56 °C for 25 s and 72 °C for 30s. The relative changes in gene expression were calculated using the equation: 2^−ΔΔCT^ [33] where all values were normalized with respect to mRNA levels of the house-keeping gene, *rpoD.* Fold changes in mRNA gene expression levels were calculated relative to untreated bacteria samples. Each real-time PCR assay was performed in triplicate from three independent experiments and the results are expressed as the mean ± SD.

**Table 1:** Genes and product function

Gene	Gene product	Gene function	Reference
*oprI*	Outer membrane lipoprotein OprI	Membrane intergrity	[46]
*oprF*	Structural outer membrane protein	Structrual stability	[44, 47]
*oprL*	Peptidoglycan lipoprotein protein OprL	Membrane integrity & biogenesis	[46]
*opr86*	Outer membrane protein BamA	Outer membrane protein assembly	[48]
*uppS*	Undecaprenyl pyrophosphate synthase	Cell wall biogenesis	[49]
*acpP*	Acyl carrier protein	Membrane biogenesis	[50, 51]
*ftsZ*	Cell division protein FtsZ	Cell division	[52, 53]
*ftsA*	Cell division protein FtsA	Cell division	[52, 53]
*ampR*	Transcriptional regulator AmpR	Virulence	[54, 55]
*lasR*	Transcriptional regulator LasR	Quorum sensing	[56, 57]

**Table 2:** Gene and primers used for qRT-PCR (sequence 5′ → 3′)

Gene	Forward primer	Reverse primer
*oprI*	GCTCTGGCTCTGGCTGCT	AGGGCACGCTCGTTAGCC
*oprF*	CTGGACGCCATCTACCACTT	CTGTCGCTGTTGATGTTGGT
*oprL*	AACAGCGGTGCCGTTGAC	GTCGGAGCTGTCGTACTCGAA
*opr86*	CGTCTACATCACCGTCAACATC	GGCGCTTCACTTCCTCTTC
*uppS*	CGGTGATCGAGGTCTGC	GGACGCTGCCAGTTCTC
*acpP*	CCATCGAAGAACGCGTTAAG	CCTGAACGGTGGTGATCTTT
*ftsZ*	GCGGTATCTCCGACATCATC	AGGTTGACGTCTTCCAGCAG
*ftsA*	CGACGAGCTGTTCACCCTGG	GCGCCTTCCATCTTCGAGGT
*ampR*	CGCGCCATCCCTTCATC	ATGTCGACGCGGTTGTTGT
*lasR*	GGACAGCCAGGACTACGAGA	ATGGACGGTTCCCAGAAAAT
*rpoD*	GCGACGGTATTCGAACTTGT	CGAAGAAGGAAATGGTCGAG

### Peptide effect on outer membrane protein expression

The outer membrane proteins (OMPs) were extracted using a method described previously with slight modifications [34]. Briefly, 0.5 L of mucoid and non-mucoid *P. aeruginosa* (1 × 10^9^ CFU/mL) was either treated with TP359 (4× of MIC) or left untreated (control) and were grown for 4 h, and centrifuged at 2000 × g (Sorvall ST 40R, ThermoFisher Scientific, Waltham, MA) for 10 min. The cell pellets each from control and treated cultures were re-suspended in 800 μl of 100 mM TrisHCl lysis buffer (0.5 M sucrose, 0.5 mM EDTA, pH 8.6) and 4.2 ml of 50 mM TrisHCl lysis buffer (pH 8.6) containing 0.5 M sucrose, 0.5 mM EDTA and 2.5 mM MgCl_2_) and incubated on ice for 15 mins, followed by centrifugation at 7000 g at 40 °C. The supernatant contained the periplasmic fraction and the cell pellets were re-suspended in 20 mM TrisHCl (pH 8.6) containing 0.5 M sucrose, 0.5 mM EDTA), followed by sonication on ice using a cycle of 40 % amplitude, for 5 min, 120 W at a frequency of 20 kHz on a Biologics Ultrasonic Homogenizer (Fischer scientific, USA). The sonicated suspension was centrifuged for 10 min at 7000 g at 40 °C to remove the cell debris, followed by ultracentrifugation (Beckman coulter, USA) at 132,000 g at 40C for 1 h to separate cytoplasmic protein fraction in the supernatant. The pellet was then re-suspended in 15 ml of 20 mM TrisHCl (pH 8.6) containing 1 % Sarkosyl and incubated for 30 min on ice and the ultracentrifugation step was repeated. Upon ultracentrifugation, outer membrane proteins were obtained as pellets and were re-suspended in 20 mM TrisHCl (pH 7.2).

### Statistical analysis

Data were analyzed by the Student’s *t*-test using Sigma Plot Software.

## Results

### Peptide characterization

**Fig. 1:**
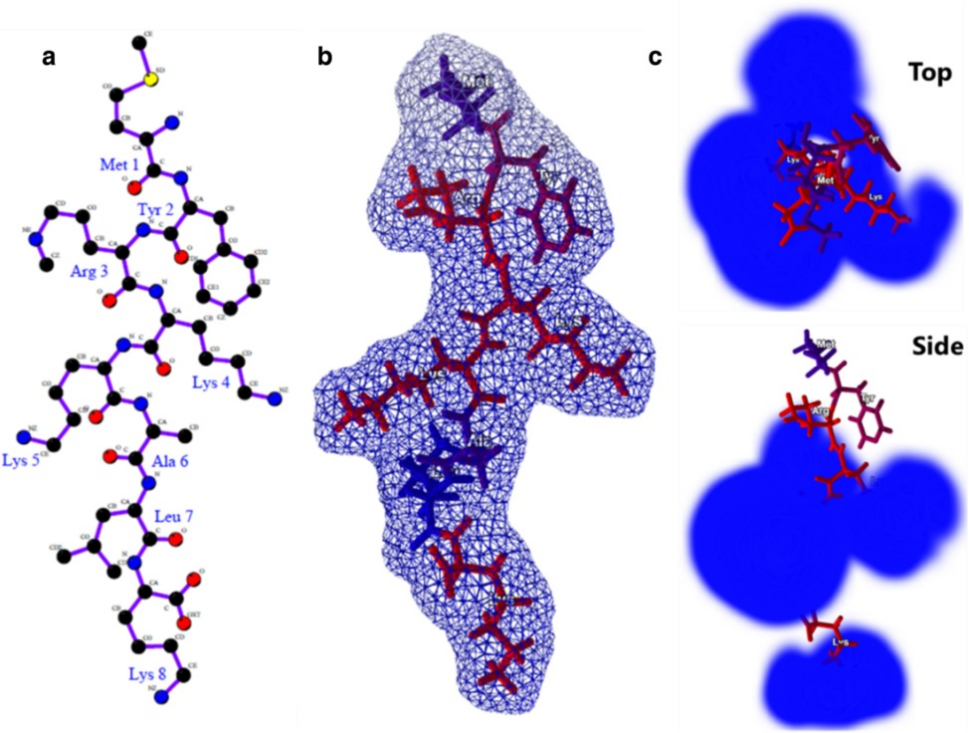
In silico analysis of TP359 **a** Structure of TP359; **b** three-dimensional structure and surface development of the peptide. The amino acids are represented as per the hydrophobicity; **c** electrostatic influence of the peptide (*top and side view*) shown in blue (positive charge)

Characterization of TP359 revealed that it is rich in the hydrophilic amino acid residue lysine, and has a net charge of +4. Grand average hydropathy value of TP359 was −1.25, indicating its hydrophilic nature, and hence its solubility in water. The TP359 peptide extends hydrophilic amino acids arginine (Arg3), and lysine (Lys4 and 5) residues towards the sides, and Lys8 towards the bottom (carboxyl end) (Fig. 1, a and b). The hydrophilic amino acid Lys contributes the positive charge to the peptide while Arg3, Lys4, Lys5 and Lys8 make it readily soluble. The non-polar amino acids at the N- terminal (aliphatic Met1 and Try2 aromatic amino acids) are followed by a polar positively charged region (Arg3, Lys4, Lys5). The C-terminal has positively charged polar Lys8; the polar, hydrophilic patches are bridged by aliphatic non-polar Ala6 and Leu7 residues. The polar and non-polar features (prominently polar) and the three-dimensional structure analyses show that the peptide may possess the ability to penetrate the cell membrane.

### TP359 is bactericidal against *P. aeruginosa*

**Fig. 2:**
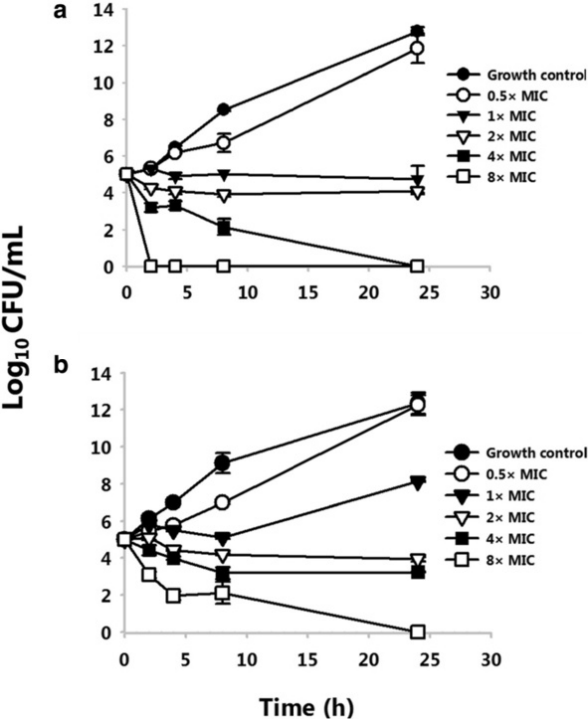
Time kill assay. Time kill assay of the mucoid (**a**) and non-mucoid (**b**) strains of *P. aeruginosa*. Killing was tested by incubating *P. aeruginosa* with peptide concentrations at the MIC, three dilutions above (2×, 4× and 8× MIC) and one dilution below the MIC (0.5× MIC) in CA-MHB. Viable cell counts were determined after 2, 4, 8 and 24 h of incubation at 37 °C. The growth control had no antibiotic. This data is a representative of three separate experiments and significance was established at *P < 0.05 and **P < 0.01

Our results show that TP359 was highly effective in preventing the growth of *P. aeruginosa*. The peptide exhibited MICs as low as 4 and 8 μg/mL and MBCs of 16 and 64 μg/mL, respectively against the mucoid and non-mucoid strains of *P. aeruginosa*. These values are consistent with the MIC range of another cationic peptide, EC5 for a different strain of *P. aeruginosa* (ATCC27835) [35]. The positive controls, polymyxin B and gentamicin sulphate displayed MICs between 0.25 and 0.5 μg/mL (Table 3). Time-kill studies were performed to determine the time-frame required for the effective killing of *P. aeruginosa* by TP359*.* Our results showed a ≥3 log CFU/mL within 8 h at 4× the MIC and a 5-log reduction of the viable cell count within 2 h at 8× MIC for the mucoid strain (Fig. 2 a). Also for the non-mucoid strain, the viable cell count decreased by ≥3 log CFU/mL within 3 h at 8× MIC (Fig. 2 b). These results demonstrate that TP359 is bactericidal against *P. aeruginosa* and that its bactericidal activity is dose- and time-dependent.

**Table 3:** Antimicrobial susceptibility of *P. aeruginosa*

MIC/MBC (μg/mL)		
	Mucoid	Non-mucoid
TP359	4/16	8/64
Gentamicin sulphate	0.5/0.5	0.25/0.25
Polymyxin B	0.5/0.5	0.5/0.5

*MIC* minimum inhibitory concentration, *MBC* minimum bactericidal concentration

### Cytotoxic activity

**Fig. 3:**
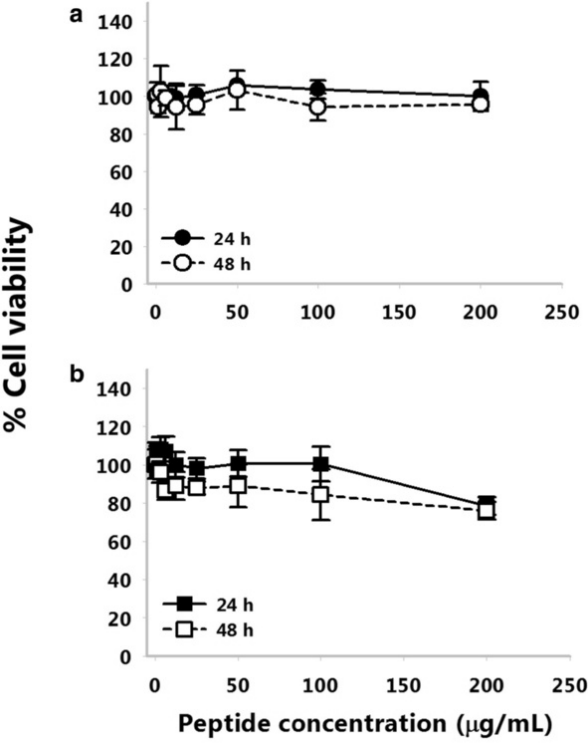
Cytotoxicity of peptide TP359. Human lung carcinoma epithelial A549 cells (**a**), and mouse J774 macrophages cells (**b**) were seeded in 96 well microtiter plates, and allowed to attach overnight before being exposed to the various peptide concentrations (0–200 μg/mL) for 24 and 48 h. Fifteen microliter of MTT dye were then added to each well and plates were subsequently incubated for 4 h before adding 100 μL of the solubilizing/stop solution and read at a wavelength of 570 nm. This data is a representative of three separate experiments and significance was established at *P < 0.05 and **P < 0.01

Given the potential consideration of TP359 as a therapeutic agent against *P. aeruginosa*, it was necessary to examine its toxicity to the human-lung carcinoma epithelial A549 and mouse J774 macrophage cell lines. Our results revealed that none of the tested concentrations (0–200 μg/mL) had toxic effects against A549 cells over a 48 h-time-frame (Fig. 3 a). TP359 was nontoxic to J774 macrophages at lower concentrations but at higher concentrations cells remained viable up to 85 % (200 μg/mL at 24 h) and (100–200 μg/mL at 48 h) (Fig. 3 b). Overall, our data suggest the safety of the peptide for potential in vivo studies.

### Membrane permeabilization

**Fig. 4:**
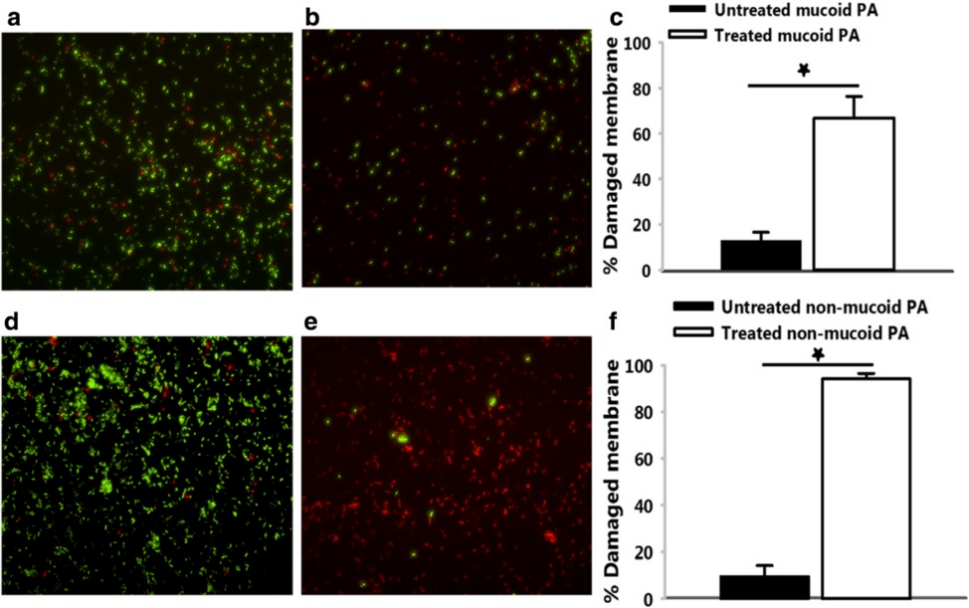
Membrane permealization. *P. aeruginosa* cells were exposed to 4× MIC of TP359 for 4 h. Cultures were centrifuged at 2000 × g for 10 min, pellets were washed, incubated and re-suspended in 0.85 % NaCl. Cells were then incubated in the dark for 45 min in a 2× stock solution of the baclight staining reagent before fluorescence was captured by the Nikon Eclipse TE200. **a** and **d** show untreated mucoid and non-mucoid *P. aeruginosa,* respectively; **b** and **e** show TP359 treated mucoid and non-mucoid *P. aeruginosa,* respectively. Images are representatives of separate experiments. For **c** and **f** random portions were selected for both treated and untreated samples using the NIS element 3.1 software and counted before extrapolating the percentage of damaged membranes in the mucoid and non-mucoid strains of *P. aeruginosa,* respectively. This data is a representative of three separate experiments and significance was established at *P < 0.05 and **P < 0.01

To begin to understand the antimicrobial mechanisms of TP359, membrane permeabilization was studied using the LIVE/DEAD stain kit, consisting of the SYTO9 and propidium iodide (PI) dyes. The SYTO9 labeled all cells (intact and permeabilized/damage membrane) green, while only damaged membranes were labelled red by PI. The percentage of cells with damaged membranes was then calculated relative to the total number of cells at different spots in different samples (Fig. 4 a – f). These data showed that TP359 is effective at permeabilizing the membranes of both strains, as the percentages of damaged membranes for both strains were ≥80 % for the treated samples, in contrast to ≤20 % for the untreated samples.

### Membrane depolarization

**Fig. 5:**
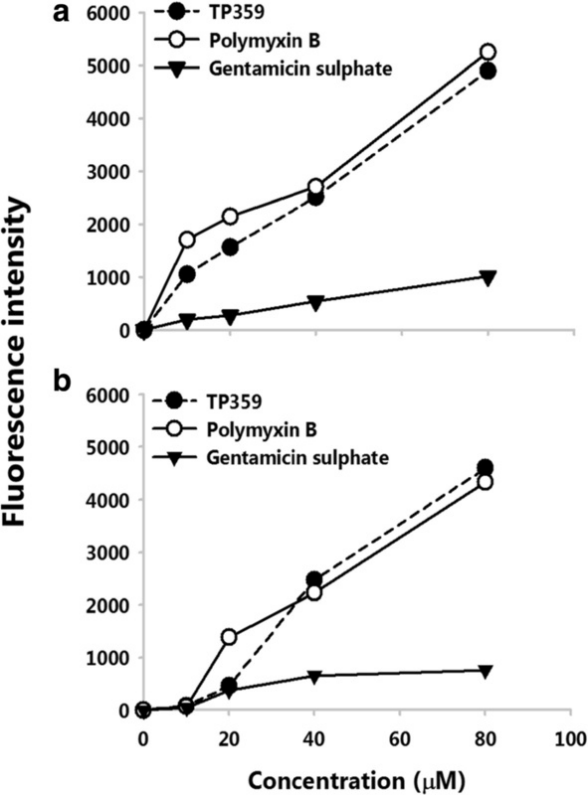
Membrane depolarization. Exponential phase bacteria of **a** mucoid *P. aeruginosa* and **b** non-mucoid *P. aeruginosa* were incubated in 0.4 mM DiSC_3_ (5). Bacterial suspensions and either peptide, polymyxin B or gentamicin sulphate were added to Optiplate 96-well to obtain a series of concentrations ranging from 10 to 80 μM. Fluorescence intensity was measured at baseline (F_i_) and after 30 min of incubation (F_f_). Increase in fluorescence intensity was then obtained by subtracting the F_i_ from F_f.._ This experiment was carried out in triplicate and significance was established at *P < 0.05 and **P < 0.01

Since results from membrane permeabilization assay indicate OM permeabilization, we sought to investigate if there is a breach of the permeability of the cytoplasmic membrane. This was performed using the DiSC_3_(5) dye which crosses the outer membrane of the bacterial cells and concentrates at the cytoplasmic membrane. Upon addition of membrane permealization agent, the dye is released into the surrounding medium, and the fluorescence intensity is measured relative to the amount of dye released. TP359 induced membrane depolarization in both strains at 10 to 80 μM was evidenced by the release of the dye. The positive polymyxin B control also induced membrane depolarization at those concentrations, while the negative gentamicin control failed to induce membrane depolarization (Fig. 5). These data show that TP359 depolarized the cytoplasmic membrane of *P. aeruginosa*, which may lead to cytoplasmic membrane damage and killing of bacteria.

### TP359 induced structural changes and damage to the membrane of *P. aeruginosa*

**Fig. 6:**
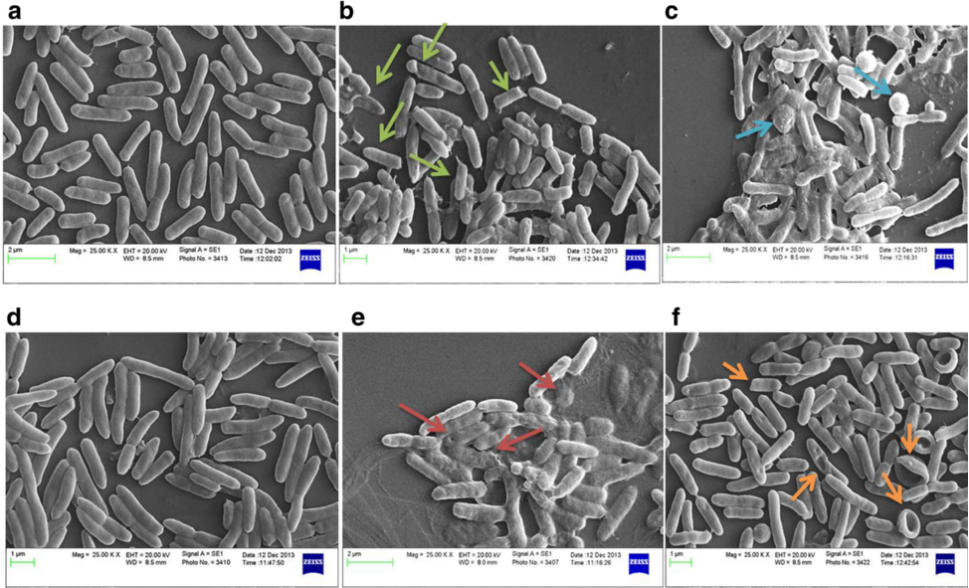
Scanning electron microscopy (SEM). TP359- treated and untreated mucoid and non-mucoid strains of *P. aeruginosa,* exposed to 4 × MIC of peptide and grown for 4 h, fixed in glutaraldehyde/paraformaldehyde, treated with osmium tetroxide, dehydrated in increasing concentrations of ethanol, and sputter coated before SEM. Panels **a** and **d** show images of untreated mucoid and non-mucoid cells respectively. Bacterial cells have intact cell membranes with uniform shape, while panels **b** and **c** show ruptured cells, with debris evident in peptide treated mucoid cells, and panels **e** and **f** show peptide treated non-mucoid bacterial cells. Treated samples show cell disintegration (*green and red arrows*), pores on cell membranes, upturned cells (*orange arrows*), and eruption of intra-cellular materials (*blue arrows*)

**Fig. 7:**
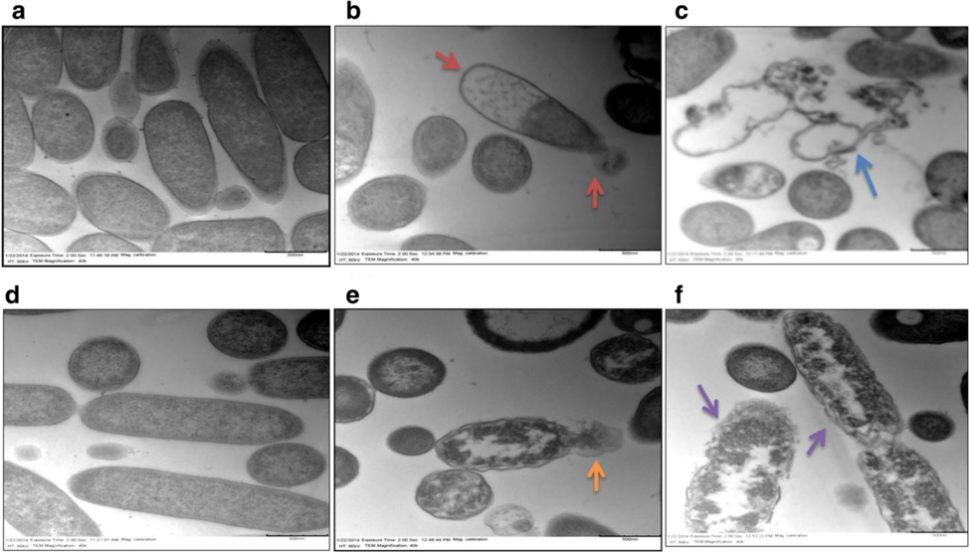
Transmission electron microscopy (TEM). *P. aeruginosa* were exposed to 4× MIC of peptide and grown for 4 h, fixed in glutaraldehyde/paraformaldehyde, treated with osmium tetroxide, dehydrated in increasing concentrations of ethanol, passed through propylene oxide, infiltrated with resin, and ultrathin sections were collected and stained with uranyl acetate and lead citrate as described in the materials and methods section. Panels **a** and **d** show images of untreated mucoid and non-mucoid cells, respectively, with intact cell membranes. Panel **b** shows peptide-treated mucoid cells with cell membrane degradation and efflux of cellular material (*red arrows*); panel **c** shows empty cells with intracellular material completely leaked out (*blue arrow*). Panel **e** shows peptide-treated non-mucoid bacterial cells with leaking intracellular material (*orange arrow*) and panel **f** shows cell membrane degradation (*purple arrow*)

We next used electron microscopy to begin to understand how TP359 kills *P. aeruginosa*. Our SEM images revealed that the TP359-treated mucoid cell population had punctured membranes, efflux of intracellular material, as well as disintegrated and agglomerated cells. Ruptured cell debris was also evident (Fig. 6, b, c), while bacteria without TP359 had smooth cell morphology (Fig. 6, a). The non-mucoid TP359-treated cell population also showed similar changes; cells were disintegrated, with evidence of damages on the membrane (Fig. 6, e, f) as compared to the untreated cells (Fig. 6, d). TEM similarly showed treated cells with compromised membranes and efflux of cytoplasmic contents proceeding into ghost cells (Fig. 7). Both SEM and TEM results established a possible mechanism of action, whereby TP359 interacts with the cell membrane causing membrane permeabilization and degradation, with resulting changes in cell morphology. These results indicate that TP359 caused loss of membrane structure resulting in morphological changes and cell death.

### TP359 effect on essential genes and protein expression of *P. aeruginosa*

**Fig. 8:**
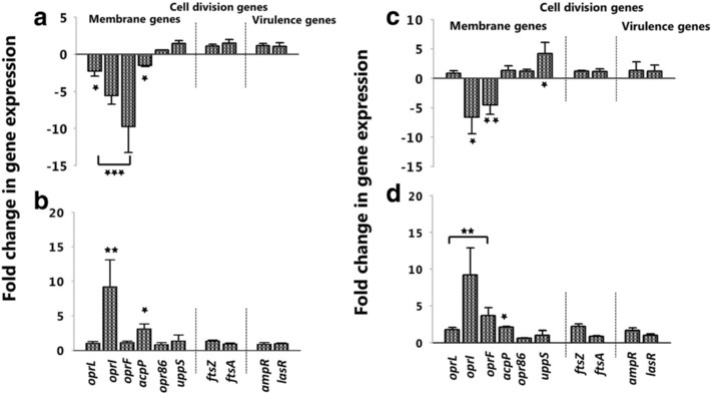
Quantitative reverse transcriptase polymerase chain reaction (qRT-PCR). *P. aeruginosa* at 1 × 10^5^ CFU/mL and 1 × 10^9^ CFU/mL were either untreated for control, or treated with 0.5× and 4× the MIC of TP359 and then grown for 18 and 4 h, representing bacteriostatic and bactericidal concentrations, respectively. Total RNA was extracted, quantified before cDNA synthesis using reverse transcriptase, followed by PCR amplification. Data obtained from three-independent experiments were used to analyze the relative gene expression by the 2^-ΔΔCt^ method. Panels **a** and **c** show the mucoid and non-mucoid strains for the bactericidal concentration and panels **b** and **d** for the bacteriostatic concentration

Selected critical genes involved in outer membrane biogenesis, peptidoglycan synthesis, cell division and virulence were analyzed to determine the molecular effect of TP359 against both strains of *P. aeruginosa* (Fig. 8). As shown, TP359 did not significantly alter expression of genes associated with cell division (*ftsA* and *ftsZ*) or virulence (*ampR* and *lasR*) under either bacteriostatic or bactericidal concentrations (Fig. 8, b and d). Contrastingly, TP359 perturbed the expression of *P. aeruginosa* membrane genes (*oprL, oprI, oprF, acpP* and *uppS)* under bacteriostatic or bactericidal concentrations (Fig. 8, b and d). Most notably, under bacteriostatic concentrations there was marked upregulation of the *oprI* gene (~10-fold) that is essential for membrane integrity in mucoid and non-mucoid strains. Examinations of the genes perturbed under bactericidal concentrations revealed a consistent and significant down-regulation of the *oprI* and *oprF* genes (Fig. 8, a and c). As shown, there was a 5-fold decrease in the expression of the *oprI* gene for both strains. However, the most notable effect of TP359 was on the *oprF* gene (essential for membrane integrity, bacterial shape, and virulence [36]), where a significant 10- and 5-fold reductions in its expression was observed for the mucoid and non-mucoid strains, respectively. This important finding shows the effect of TP359 on the *oprF* gene, which may suggest that disruption of membrane integrity, could contribute to bacterial cell death. Moreover, this result strongly supports the findings of the SEM and TEM analysis, where TP359-treated cells show flaccid membranes, compared to the untreated control cells. Additionally, *P. aeruginosa* exposed to TP359 for 10 and 30 mins showed no significant changes when compared to the untreated, indicating that a longer period of treatment was required to evaluate TP359 effect on these genes (Additional file 1: Figure S1). Also, *P. aeruginosa* exposed to gentamicin showed no significant changes when compared to the TP359 treated samples, suggesting that TP359 effect was not merely due to stress (Additional file 2: Figure S2).

**Fig. 9:**
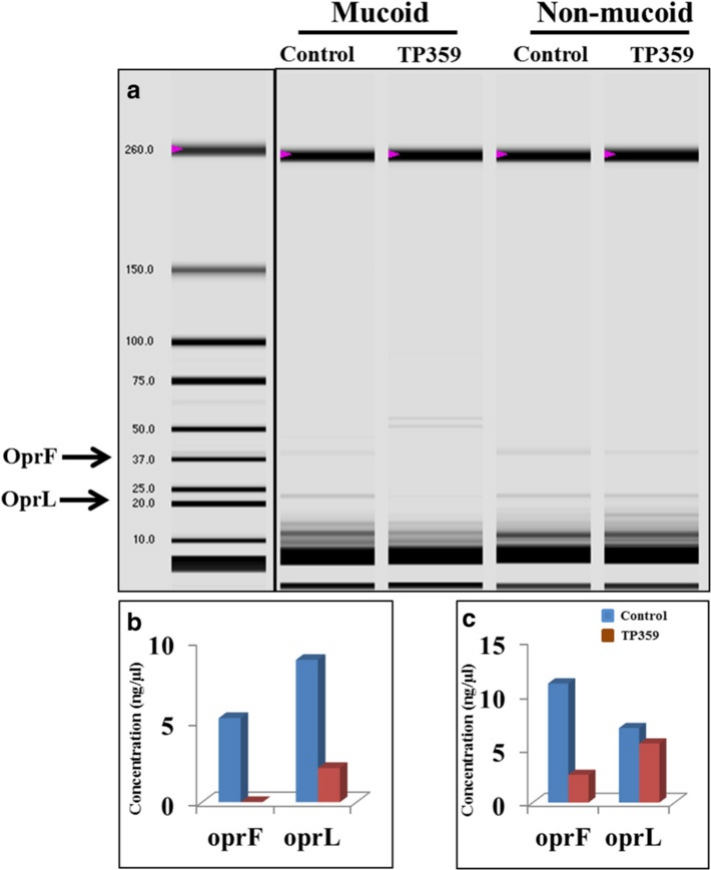
Evaluation of outer membrane proteins of TP359 –treated and untreated *P. aeruginosa. P. aeruginosa* at 1 × 10^9^ CFU/mL were either untreated for control, or treated with 4× the MIC of TP359 and grown for 4 h. The OMP fraction was extracted and then separated on the Experion^TM^ Pro260 automated electrophoresis system for analysis. Panel **a** show the protein concentrations of OprF and OprL (as analyzed by the automated system) and **b** and **c** represent the mucoid and non-mucoid strains respectively

We confirmed the effect of the peptide on outer membrane proteins by analyzing outer membrane protein fractions of TP359-treated *P. aeruginosa* using the Experion^TM^ automated electrophoresis system (Fig. 9 a) followed by semi-quantification of proteins (Fig. 9 b and c). As shown in Fig. 9 a, there was a fully expressed 37.6 kDa OprF protein in both untreated mucoid and non-mucoid *P. aeruginosa* samples. In contrast, expression was visually absent in the TP359-treated mucoid strain and reduced in the TP359-treated non-mucoid strain samples. This data correlated with our gene expression analysis, where a significant down-regulation of the *oprF* gene was observed for both strains of *P. aeruginosa*. The OprL protein expression also was significantly reduced in the TP359-treated samples as compared to the untreated samples for the mucoid (Fig. 9 a and b) but not the non-mucoid strain (Fig. 9 a and c), which is congruent with the gene expression results. The OprI, with molecular weight less than 10 KDa was undetectable because its size is below the detection limit for the Experion^TM^ automated electrophoresis system.

## Discussion

The antimicrobial activity and molecular effects of a novel proprietary cationic peptide, TP359 were investigated independently against a mucoid and a non-mucoid strain of *P. aeruginosa*. Our results revealed the following: (i) both strains of *P. aeruginosa* were sensitive to TP359, (ii) TP359 showed no toxic effects against human lung epithelial A549 and mouse J774 macrophage cell lines, (iii) TP359 caused membrane damage, as shown by SEM, TEM, LIVE/DEAD stain and membrane depolarization assay and (iv) TP359 down-regulated *P. aeruginosa* membrane biogenesis and peptidoglycan synthesis associated genes.

TP359 exhibited antimicrobial activity against both strains of *P. aeruginosa* at MIC concentrations, which is in agreement with most cationic AMPs [35, 37], with comparatively lesser effect on Gram-positive strains. It was essentially non-toxic to eukaryotic cells, which is also in agreement with other AMPs [38–40]. The lack of toxicity of AMPs to eukaryotic cells may be due to their membrane composition, which consists of zwitterionic phospholipids that are neutral in net charge [6]. This neutral charge reduces electrostatic interaction between the eukaryotic cell membrane and AMPs, thus increasing their potential for use as antipseudomonal drugs. Our investigation into the effect of TP359 on the membrane of *P. aeruginosa* revealed that TP359 caused outer membrane permeabilization, which was further illustrated in the TP359 sensitivity assay, where bacterial cells were sensitive to TP359 (data not shown), suggesting its effect on cell membrane integrity. Generally, the antimicrobial activity of AMPs is mostly attributed to membrane dysfunction, and cationic amphiphilic AMPs are electrostatic, which makes them attracted to the negatively charged bacteria membrane surfaces [13, 26] causing membrane damage. Results from our SEM and TEM analyses further confirm this, together with the leaking of cytoplasmic content. These observations suggest that TP359 may penetrate the bacterial membrane by electrostatic interaction between TP359 and the bacterial cell surface.

Although the classic mechanism of action of AMPs is their ability to cause membrane damage, other mechanisms involve the inhibition of cell wall biogenesis, such as the lantibiotics-class 1 bacteriocins, Lcn 972, a class 2 bacteriocin and nicin [41]. Another class of peptides which target cell wall biogenesis is the class of endogenous host defense peptides (HDP) such as the defensins and cathelicidins [42]. Some reports also indicate that membrane perturbation alone may not be sufficient to elicit bacterial cell death [8, 43], so we investigated the molecular effect of TP359 against selected critical genes involved in membrane biogenesis, peptidoglycan synthesis associated genes, cell division genes and virulence genes.

Analysis of TP359-treated cells showed that the transcriptional expression levels of some outer membrane genes were down-regulated at bactericidal concentrations, whereas they were up-regulated at bacteriostatic concentrations in both strains. The most significantly down-regulated gene in both strains at bactericidal concentrations was *oprF,* which encodes a major structural and outer membrane porin (OprF). The reduced translational expression of OprF further underscores its dysregulation by TP359. *P. aeruginosa* OprF is essential for cell shape, maintenance of peptidoglycan association and structural integrity [44] and also for complete virulence. Therefore, a down-regulation of OprF expression, suggests a disruption in the outer membrane of the bacterial cell, which could result into a permeable membrane for foreign materials and water, leading to osmosis and cell lysis. This effect of the peptide on oprF expression indeed correlates with our SEM analysis, where TP359-treated cells had a rough shape compared to the untreated cells, indicating defective bacteria membrane integrity. The significance of our findings is further accentuated by the observation that *oprF* mutants formed poor anaerobic biofilms in the lung of CF patients, and that lung secretions from CF patients with chronic *P. aeruginosa* infection have increased antibodies to OprF [45], indicating the significance of OprF for persistent infection. Hence, based on our findings, we could infer that TP359 could disrupt the cellular integrity of *P. aeruginosa* and increase clearance from CF airways. As a positive control, the effect of gentamicin on the outer membrane genes was investigated at both bactericidal and bacteriostatic condition. Although some of the genes were downregulated in both treated strains, they were not statistically significant, suggesting that the effect of TP359 on these genes may not be due to stress and could be a contributory factor to its antimicrobial activity.

**Fig. 10:**
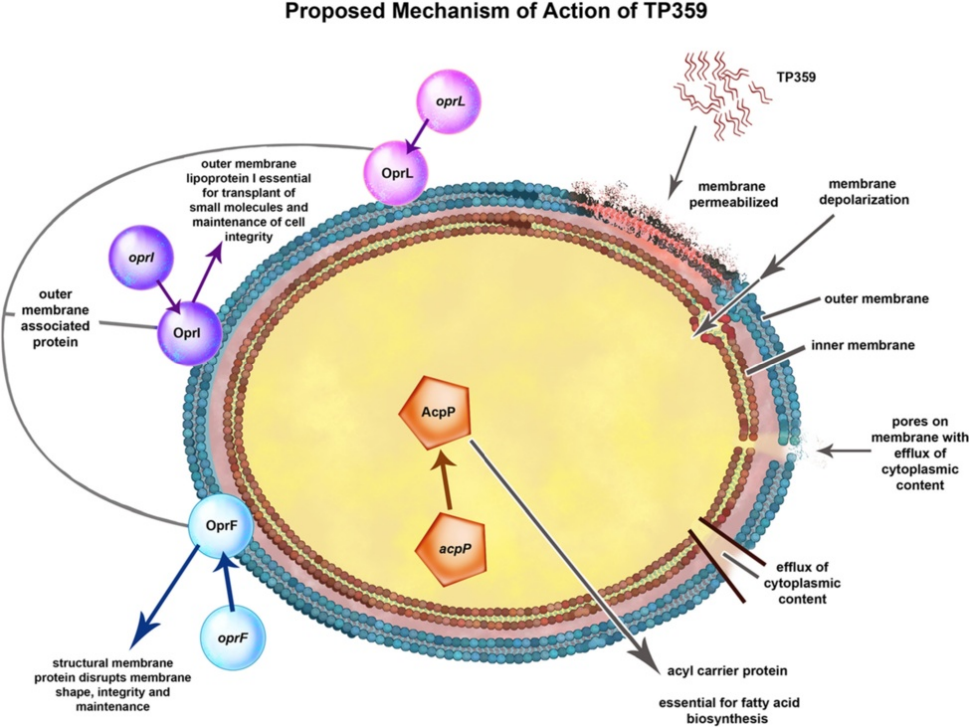
Proposed mechanism of action of TP359. The proposed mechanism of action involves the interaction of the cationic TP359 with the outer membrane of *P. aeruginosa*, which is negatively charged due to lipopolysaccharide (LPS), causing membrane permeabilization and depolarization. This causes pores on the membrane resulting in efflux of cytoplasmic content to produce ghost cells. Furthermore, TP359 also caused down-regulation of outer membrane associated genes such as the *acpP*, *oprL*, *oprI* and most importantly the *oprF*. The protein products, AcpP, OprL, OprI and OprF are essential for fatty acid biosynthesis, transport of small molecules and structural membrane integrity. The effect of TP359 on the *oprF* gene may disrupt its product, the OprF, which encodes membrane integrity and structure, leading to a compromised membrane and ultimately cell death

To our knowledge, our study is the first to comprehensively characterize the antimicrobial effect of a cationic AMP at the gene level. Based on the novelty of our findings, we propose the possible mechanisms of action of the TP359 peptide (Fig. 10) which may include: i) direct contact with the bacterial cell surface due to the attraction between the negatively charged bacterial membrane and the positively charged peptide ii) permeabilization of the outer membrane either due to the downregulation of the outer membrane gene *oprF,* which is essential for maintaining membrane integrity and shape; or the presence of the hydrophilic portion of the peptide, in the hydrophobic rich portion of the bacterial membrane resulting in membrane instability, in addition to other outer membrane biogenesis genes and iii) inner membrane depolarization, through the displacement of membrane charges.

## Conclusion

Our novel cationic peptide TP359 showed antibacterial activity against both strains of *P. aeruginosa*. TP359 also induced significant morphological changes, and reduced expression of outer membranes and peptidoglycan associated genes specifically the *oprF* gene, suggesting that the *oprF* gene could be a target for antimicrobial drug development. Since the use of polymyxin B to treat *P. aeruginosa* has been abandoned due to its nephrotoxicity, TP359, which showed low cytotoxicity in vitro, may prove to be a valuable therapeutic alternative, especially for CF patients. Further studies would include the study of other cationic AMPs and verification of their effect on outer membrane biogenesis genes, which could enhance targeted antimicrobial effects. These could lead to in vivo investigations of the antimicrobial activity of TP359.

## Additional files

Additional file 1: Figure S1. Differential gene expression levels of selected genes for the mucoid and non-mucoid strains of *P. aeruginosa* exposed to TP359 bactericidal treatment*. P. aeruginosa* at 1 × 10^9^ CFU/mL were either untreated as control, or treated with 4× the MIC of TP359 and then grown for 10 and 30 min. Total RNA was extracted, quantified before cDNA synthesis using reverse transcriptase, followed by PCR amplification. Data obtained from three-independent experiments were used to analyze the relative gene expression by the 2^-ΔΔCt^ method. Panels A and B show the 10 and 30 min treatment for the mucoid strain and non-mucoid strain, respectively. (JPG 145 kb) Additional file 2: Figure S2. Differential gene expression levels of selected genes for the mucoid and non-mucoid strains of *P. aeruginosa* exposed to gentamicin bactericidal treatment. *P. aeruginosa* at 1 × 10^9^ CFU/mL were either untreated for control, or treated with 4× the MIC of gentamicin and then grown for 4 h. Total RNA was extracted, quantified before cDNA synthesis using reverse transcriptase, followed by PCR amplification. Data obtained from three-independent experiments were used to analyze the relative gene expression by the 2-ΔΔCt method. Panels A and B show the gentamicin treatment for the mucoid and non-mucoid strains of *P. aeruginosa*, respectively. (JPG 83 kb)

## Abbreviations

AMPs: Antimicrobial peptides
CA-MHB: Cation-Adjusted Mueller Hinton broth
cDNA: Complementary deoxyribonucleic acid
CF: Cystic fibrosis
CFU/mL: Colony forming unit per milliliter
CLSI: Clinical and laboratory standards institute
DMEM: Dulbecco’s modified eagle medium
FBS: Fetal bovine serum
HDP: Host defense peptide
LB: Luria-Bertani medium
MBC: Minimum bactericidal concentration
MIC: Minimum inhibitory concentration
mRNA: Messenger ribonucleic acid
PB: Polymyxin B
PCR: Polymerase chain reaction
PI: Propidium iodide
qRT-PCR: Quantitative real time-polymerase chain reaction
SEM: Scanning electron microscopy
TEM: Transmission electron microscopy
THY: Todd-Hewitt medium supplemented with yeast extract
UTI: Urinary tract infection
