# Supplementary figures and images for: Novel cationic peptide TP359 down-regulates the expression of outer membrane biogenesis genes in Pseudomonas aeruginosa: a potential TP359 anti-microbial mechanism
Source: BMC Microbiol. 2016 Aug 22;16(1):192. doi: 10.1186/s12866-016-0808-2 (PMC4994277; doi:10.1186/s12866-016-0808-2)

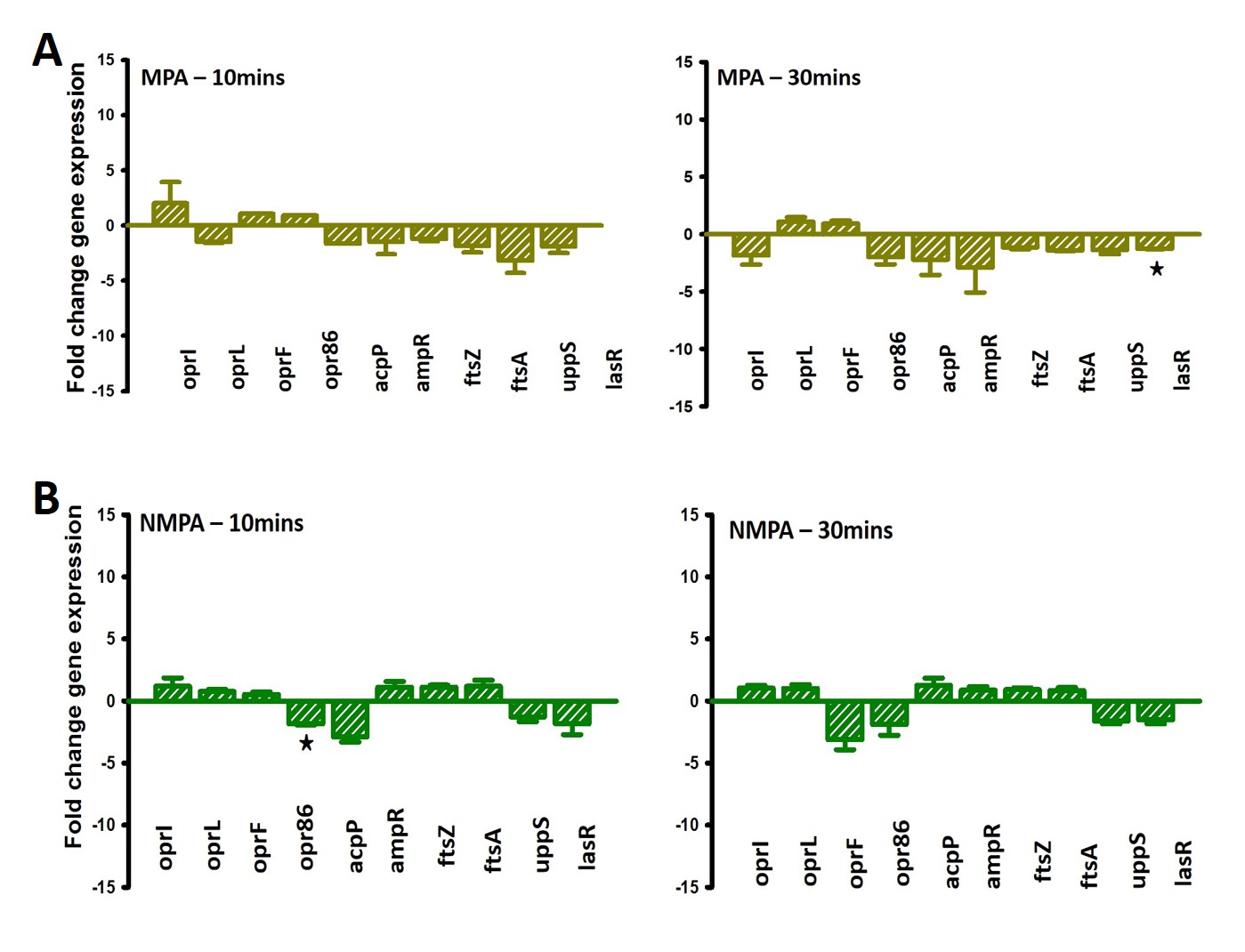

Supplement: Additional file 1: Figure S1. — Differential gene expression levels of selected genes for the mucoid and non-mucoid strains of P. aeruginosa exposed to TP359 bactericidal treatment. P. aeruginosa at 1 × 10^9^ CFU/mL were either untreated as control, or treated with 4× the MIC of TP359 and then grown for 10 and 30 min. Total RNA was extracted, quantified before cDNA synthesis using reverse transcriptase, followed by PCR amplification. Data obtained from three-independent experiments were used to analyze the relative gene expression by the 2-ΔΔCt method. Panels A and B show the 10 and 30 min treatment for the mucoid strain and non-mucoid strain, respectively. (JPG 145 kb) [file 12866_2016_808_MOESM1_ESM.jpg]

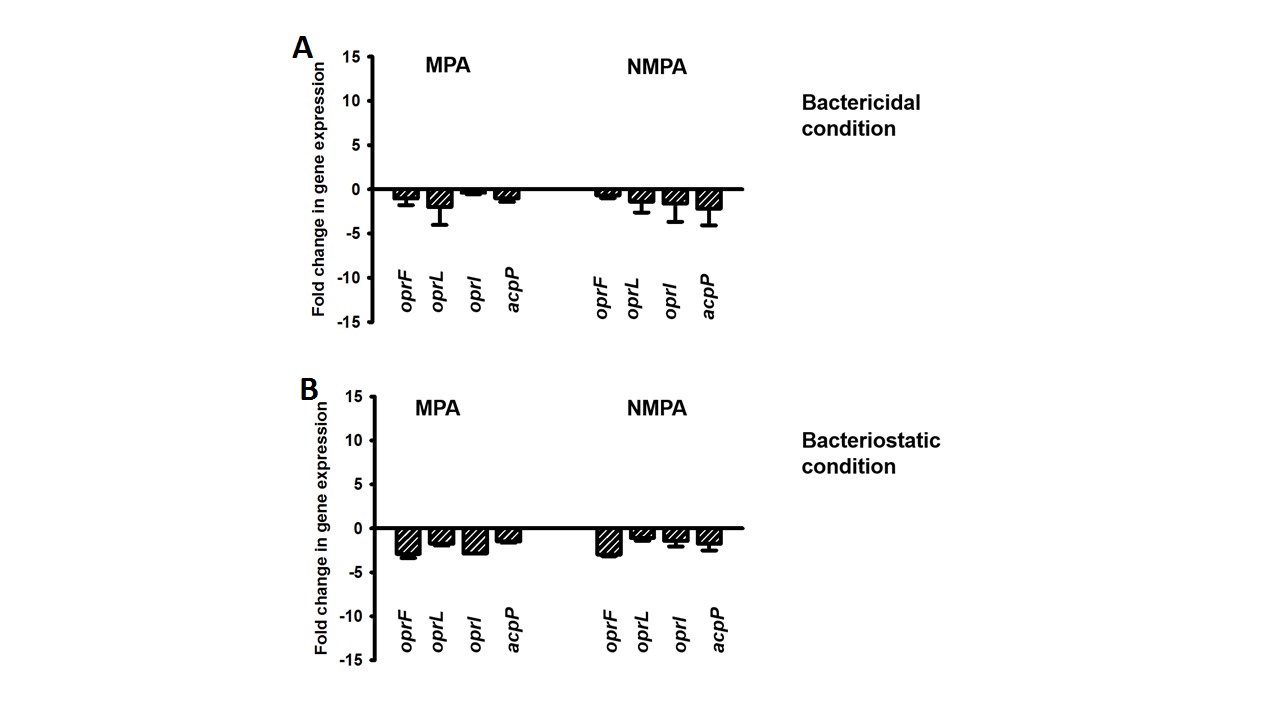

Supplement: Additional file 2: Figure S2. — Differential gene expression levels of selected genes for the mucoid and non-mucoid strains of P. aeruginosa exposed to gentamicin bactericidal treatment. P. aeruginosa at 1 × 10^9^ CFU/mL were either untreated for control, or treated with 4× the MIC of gentamicin and then grown for 4 h. Total RNA was extracted, quantified before cDNA synthesis using reverse transcriptase, followed by PCR amplification. Data obtained from three-independent experiments were used to analyze the relative gene expression by the 2-ΔΔCt method. Panels A and B show the gentamicin treatment for the mucoid and non-mucoid strains of P. aeruginosa, respectively. (JPG 83 kb) [file 12866_2016_808_MOESM2_ESM.jpg]
